# Resolution of habitat-associated ecogenomic signatures in bacteriophage genomes and application to microbial source tracking

**DOI:** 10.1038/s41396-017-0015-7

**Published:** 2017-12-19

**Authors:** Lesley A. Ogilvie, Jonathan Nzakizwanayo, Fergus M. Guppy, Cinzia Dedi, David Diston, Huw Taylor, James Ebdon, Brian V. Jones

**Affiliations:** 1 School of Pharmacy and Biomolecular Sciences, University of Brighton, Brighton, UK; 2 Mikrobiologische & Biotechnologische Risiken Bundesamt für Gesundheit BAG, 3003 Bern, Switzerland; 3 School of Environment and Technology, University of Brighton, Brighton, UK

## Abstract

Just as the expansion in genome sequencing has revealed and permitted the exploitation of phylogenetic signals embedded in bacterial genomes, the application of metagenomics has begun to provide similar insights at the ecosystem level for microbial communities. However, little is known regarding this aspect of bacteriophage associated with microbial ecosystems, and if phage encode discernible habitat-associated signals diagnostic of underlying microbiomes. Here we demonstrate that individual phage can encode clear habitat-related 'ecogenomic signatures', based on relative representation of phage-encoded gene homologues in metagenomic data sets. Furthermore, we show the ecogenomic signature encoded by the gut-associated ɸB124-14 can be used to segregate metagenomes according to environmental origin, and distinguish 'contaminated' environmental metagenomes (subject to simulated in silico human faecal pollution) from uncontaminated data sets. This indicates phage-encoded ecological signals likely possess sufficient discriminatory power for use in biotechnological applications, such as development of microbial source tracking tools for monitoring water quality.

## Introduction

The faecal contamination of environmental waters used for drinking and recreational purposes poses a major potential risk to public health. Detection of faecal contamination and determination of its origin (microbial source tracking; MST) is an emerging element in managing these risks and safeguarding water quality. At present, the cultivation of faecal indicator bacteria (FIB) from water samples, such as faecal coliforms, *Escherichia coli* and *Enterococcus* spp., remains a mainstay of methods for detecting faecal pollution of water resources [1–4]. Although the detection and enumeration of FIB have long been useful in strategies to improve and maintain water quality, they are subject to a range of limitations that impair their overall utility. Limitations include their lack of specificity to human faeces, poor persistence or potential regrowth in certain environments, and long turnaround times associated with culture-based detection [5–8].

Consequently, numerous alternative human-specific MST approaches have been developed in recent years, including both culture-dependent and molecular-based approaches. Culture-independent, molecular-based approaches to MST are increasingly attractive as they offer the potential to overcome certain limitations inherent in culture-dependent approaches. These include a reduced turnaround time and improved sensitivity, which should lead to more efficient quantification and prediction of risk. Ultimately, molecular-based MST approaches could conceivably deliver an indication of water quality directly at the point of sample collection, and in near real time [9].

To date, the development of molecular-based MST methods has focused primarily on the detection and amplification of target genes or sequences associated with specific faecal bacteria, typically using either end point or quantitative PCR [3, 10]. More recently, improved access to high-throughput next-generation sequencing technologies, along with the growing portability, ease-of-use and affordability of such systems, have begun to offer the prospect of developing metagenomic approaches to MST (e.g., refs. [9, 11]). The application of metagenomics to MST should permit high-resolution methods based on surveillance of whole microbial communities, and identification of habitat-specific genetic patterns that can distinguish microbial ecosystems (also termed 'ecogenomic signatures').

Alternatives to FIB are also likely to be important in the development of more effective MST tools. In particular, the detection of human gut-specific bacteriophage (phage) that infect anaerobic gut bacteria are increasingly viewed as potentially superior indicators of pollution compared to direct detection of their bacterial host. The advantages of phage for MST are a longer environmental persistence, greater abundance than the host bacteria and the ability of phage to replicate within cultured host species. All of which can serve to amplify any signal of human faecal contamination and improve sensitivity [12, 13]. These potential advantages of phage in MST are further supported by reports of the isolation and characterisation of apparently human gut-specific phage, and the subsequent use of these as MST tools [12–17].

Furthermore, many of the advantages offered by phage in traditional culture-based MST methods [15] would also seem to apply to the development of phage-based culture-independent approaches. These include metagenomic MST tools, which could conceivably target the entire retinue of viruses associated with a particular microbial ecosystem (the virome). However, the potential for such virome-based metagenomic MST is currently uncertain, and first requires fundamental study in order to define the principles under which phage-based metagenomic MST could operate. In particular, it remains unclear to what extent individual phage, or wider phage communities, associated with target ecosystems are diagnostic of underlying host microbiomes and contain unambiguous ecogenomic signals which offer sufficient discriminatory power for MST.

Here we hypothesise that individual human gut-associated phage, infecting key members of this microbiome, will encode a distinct habitat-associated signal derived from the co-evolution and adaptation of phage and host to life within the human gut. If so, homologues of genes encoded by such phage should display an increased relative abundance in human gut-derived metagenomes, compared to metagenomes from other microbial ecosystems. To test these theories, we utilised publically available viral and whole community metagenomic data sets to develop a comprehensive ecological profile of ɸB124-14, a phage previously proven to infect a restricted set of human-associated *Bacteroides fragilis* strains, including those with MST utility [18, 19], and compared this to phage from non-gut habitats. Our previous genetic and ecological profiling of ɸB124-14, indicated that this phage has utility as a marker of human faecal pollution, with potential as a platform for the development of quantitative molecular MST tools [18]. As such, ɸB124-14 constitutes an excellent model with which to begin to explore the existence of habitat-specific ecogenomic signatures in phage genomes and their application to development of improved MST approaches.

## Results

### Representation of sequences with similarity to bacteriophage-encoded ORFs in viral metagenomes

**Fig. 1:**
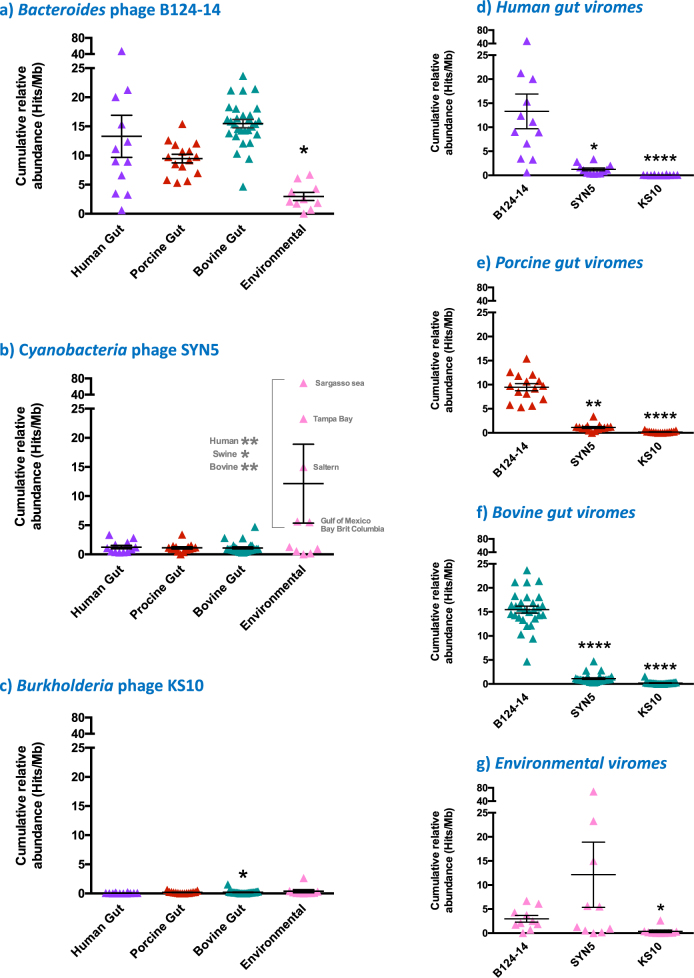
Cumulative relative abundance of sequences with similarity to ORFs encoded by *Bacteroides* ɸB124-14, *Cyanophage* SYN5 and *Burkholderia* phage KS10 in viral metagenomes. Reads from each virome were mapped to translated ɸB124-14, ɸSYN5 or ɸKS10 ORFS using BlastX. Details of data sets used are provided in Supplementary Table S1. **a**–**c** Relative representation of phage ORFs across habitats represented by viromes. Charts show cumulative relative abundance of sequences with similarity to ORFs encoded by *Bacteroides* ɸB124-14, *Cyanobacteria* ɸSYN5 and *Burkholderia* ɸKS10. For environmental data sets, those derived from temperate marine environments most relevant to the predicted ɸSYN5 host habitat were also analysed as a distinct subgroup. **d**–**g** Comparison of phage representation within specific habitats. Charts show cumulative relative abundance of sequences with homology to ORFs from each phage examined in viral metagenomes from the human gut, porcine gut, bovine gut and the environment. In all figures, bars show mean plus SEM and statistically significant differences denoted by *P ≤ 0.05, **P ≤ 0.01 ****P ≤ 0.0001 vs. human gut viromes (**a**–**c**) or ɸB124-14 (**d**–**g**)

To evaluate the relative representation of genes with similarity to those encoded by ɸB124-14 in viral metagenomes, we calculated the cumulative relative abundance of sequences similar to translated ɸB124-14 open reading frames (ORFs) in each metagenome (Fig. 1). These data sets encompassed the human, porcine and bovine gut, as well as a broad range of aquatic environmental habitats (see Supplementary Table S1). Sequences generating valid hits to at least one ɸB124-14 ORF were identified in all data sets evaluated, but a significantly greater mean relative abundance of ɸB124-14-encoded ORFs was evident in human gut viromes, compared with environmental data sets (Fig. 1a). No significant differences were apparent between the mean cumulative relative abundance of ɸB124-14 human gut viromes and other gut viromes examined (Fig. 1a). Individual human gut viromes were also observed to display a notably greater variation in ɸB124-14 cumulative relative abundance than other data sets analysed (Fig. 1a).

To determine if these 'gut-associated' ɸB124-14 relative abundance profiles represented a habitat-related signal in ɸB124-14, or could be attributed to properties of phage genomes or the human gut virome in general, we repeated this experiment using additional genomes from phage not considered to be associated with the human gut. These included the *Cyanophage* SYN5 [20], and the *Burkholderia* prophage KS10 [21]. ɸSYN5 was isolated from temperate marine environments, while ɸKS10 was identified in *B. cenocepacia* strain K56-2, an organism typically associated with the plant rhizosphere, but also an opportunistic human pathogen [22]. Based on tetranucleotide profiling, ɸKS10 has previously been shown to be among the most distantly related phage to ɸB124-14 [18].

Neither ɸSYN5 nor ɸKS10 exhibited the gut-associated enrichment of similar ORFs evident for ɸB124-14, when cumulative relative abundance profiles of each phage were considered across all habitats represented (Fig. 1b, c). However, ɸSYN5 displayed a significantly greater representation in a subset of data sets from marine environments relative to gut viromes, congruent with its environmental origin and indicative of an ecological profile distinct from ɸB124-14 (Fig. 1b). In contrast, sequences similar to ɸKS10 ORFs appeared to be only very poorly represented in the majority of data sets examined, with no discernible ecogenomic profile identified within the data sets analysed (Fig. 1c). Comparison of phage-to-phage relative abundance profiles within specific habitats reinforced the potential for a gut-associated ecogenomic signal in ɸB124-14, with ɸSYN5 and ɸKS10 shown to have significantly lower representation in all gut-derived viromes examined (Fig. 1d, e, f, g).

### Detection of the ɸB124-14 ecogenomic signal in whole community metagenomes

Because the human gut virome is believed to be dominated by temperate phage [23, 24], and we have previously demonstrated that conventional whole community shotgun metagenomes derived from human gut bacteria capture notable fractions of the gut-associated *Bacteroides* phage population [25], we next explored the representation of ɸB124-14 ORFs in assembled whole community metagenomes. These encompassed data sets derived from the human gut and other body sites, as well as a range of non-human gut and environmental habitats (Supplementary Table S1).

**Fig. 2:**
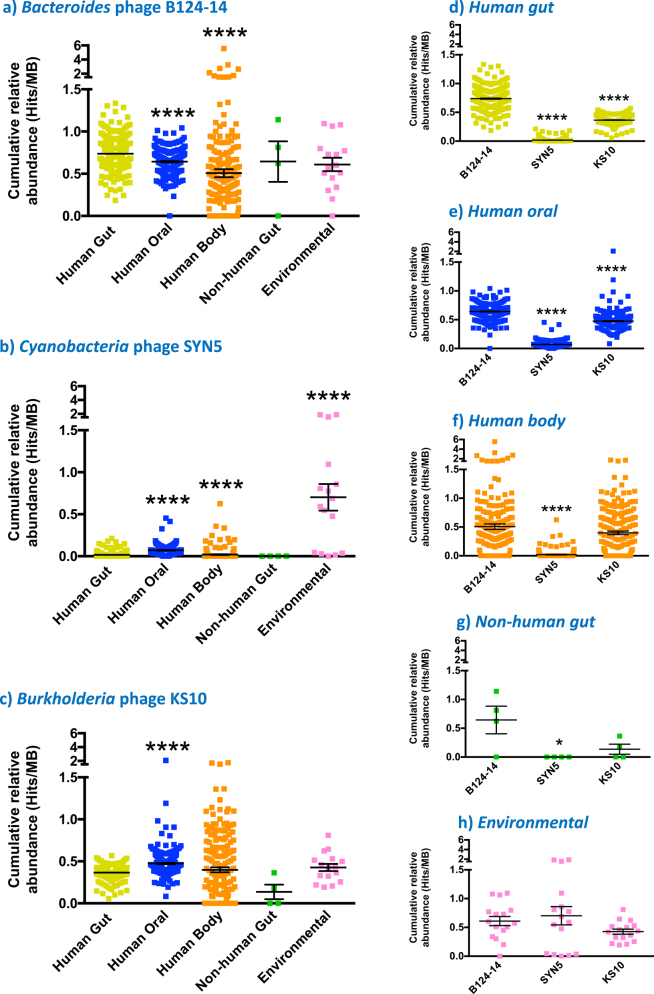
Cumulative relative abundance of sequences with similarity to ORFs encoded by ɸB124-14, *Cyanophage* SYN5 and *Burkholderia* phage KS10 in assembled whole community metagenomes. Data sets were searched using translated ɸB124-14, ɸSYN5 or ɸKS10 ORF sequences using tBlastn. Valid hits were used to calculate the cumulative relative abundance sequences with similarity to phage ORFs in each data set (expressed as Hits/Mb). **a**–**c** Relative representation of phage ORFs across habitats represented by whole community metagenomes. Charts show cumulative relative abundance of sequences with similarity to ORFs encoded by *Bacteroides* ɸB124-14, *Cyanobacteria* ɸSYN5 and *Burkholderia* ɸKS10. **d**–**g** Comparison of phage representation within specific habitats. Charts show cumulative relative abundance of sequences with similarity to ORFs from each phage examined in whole community metagenomes from the human gut, human oral cavity (mouth and throat), other human body sites (skin, nares and vagina), non-human gut and wider environment. For all data sets, bars show mean plus SEM. ***P < 0.001, ****P < 0.0001 vs. environmental viromes (**a**–**c**) or ɸB124-14 (**d**–**g**)

Analysis of the cumulative relative abundance of sequences with similarity to ɸB124-14 ORFs across habitats, showed no significant differences between whole community human gut metagenomes and non-human gut or environmental data sets (Fig. 2a). A significantly decreased representation at other human body sites compared to the human gut was detected (Fig. 2a). Identical analyses using ɸSYN5 showed that, compared to human gut data sets, ɸSYN5 ORFs had significantly greater representation in environmental data sets, congruent with the environmental origin of this phage (Fig. 2b). ɸKS10 again showed no discernible ecological profile within these data sets (Fig. 2c).

When phage relative abundance profiles were compared directly within specific habitats on a phage-to-phage basis, a significantly greater representation of sequences with similarity to ɸB124-14 ORFs was apparent in human-derived data sets in general, compared with ɸSYN5 or ɸKS10 (Fig. 2d, e, f). ɸB124-14 ORFs also showed significantly greater representation in non-human gut metagenomes compared to ɸSYN5 (Fig. 2g), but no significant differences were noted between phage when environmental metagenomes were examined (Fig. 2h).

### The ɸB124-14 ecogenomic signal can discriminate human gut viromes from other data sets

Given the observed enrichment of sequences with similarity to ɸB124-14 ORFs in mammalian gut-derived viral metagenomes, and other human-derived whole community metagenomes, we next examined the potential for this putative ecogenomic profile to distinguish human gut metagenomes from those derived from other habitats. We reasoned that a genuine habitat-related ecogenomic signature should permit the accurate segregation and grouping of metagenomic data sets based on their environmental origin. To test this, non-metric multidimensional scaling (nMDS) was used for unsupervised ordination of individual metagenomes, based on relative abundance profiles of ɸB124-14 ORFs in each data set. The level and significance of separation between groups of metagenomes was subsequently investigated using analysis of similarities (ANOSIM) [26]. To increase stringency-only metagenomes with representation of at least two distinct phage ORFs were included in this analysis.

**Fig. 3:**
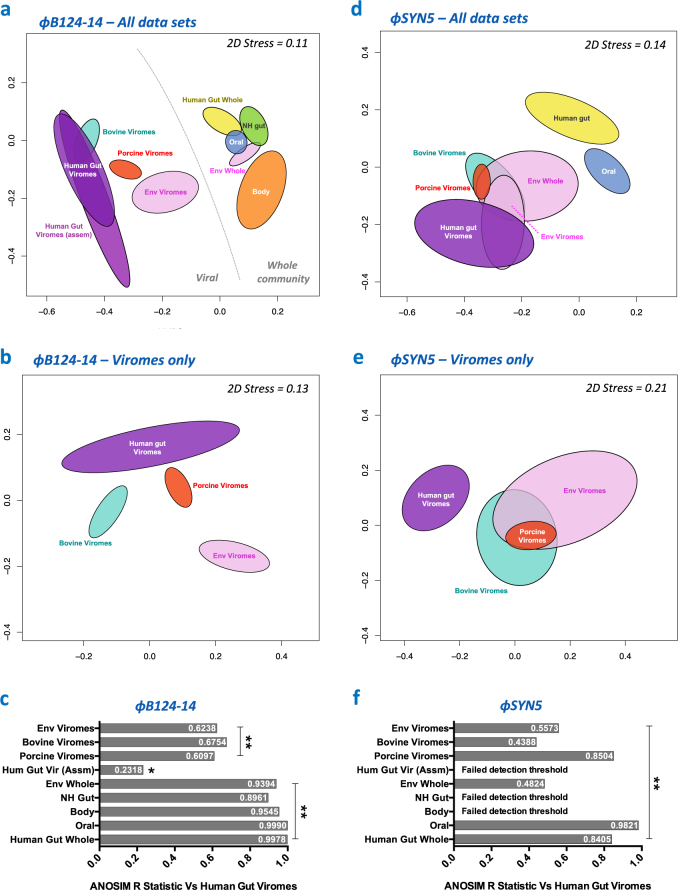
Unsupervised ordination of metagenomic data sets based on phage ecogenomic signatures. Non-metric multidimensional scaling (nMDS) was used to ordinate individual metagenomic data sets based on the relative abundance profiles of individual ORFs from either ɸB124-14 or ɸSYN5. The strength and significance of separation between groups of metagenomes with related environmental origins was evaluated using ANOSIM. To reduce the noise and increase stringency, only metagenomes with representation of two or more distinct phage ORFs were included in this analysis. **a**, **b**, **d**, **e** nMDS ordination of all metagenomes (all data sets), or exclusively viral metagenomes (viromes only), based on ɸB124-14 or ɸSYN5 ORF relative abundance profiles. Filled ellipses show standard deviation of dispersion of each group relative to the group centroid. For nMDS based on ɸSYN5 relative abundance profiles, no data sets from human gut virome assemblies, human oral cavity or human body sites met the minimum criteria for inclusion. **c**, **f** ANOSIM analysis of differences between groups of metagenomes used in nMDS. Charts show the ANOSIM R statistic for each comparison relative to the unassembled human gut viral data sets. An increasing strength of separation between groups is indicated as the R statistic approaches 1 (total separation). Symbols above bars indicate statistical significance of observed separation between groups: **P ≤ 0.001, *P ≤ 0.05. For ɸSYN5 analyses, groups where no data sets met the threshold criteria for representation of a minimum of two distinct ORFs, were not included in nMDS or ANOSIM and indicated as 'failed detection threshold' in **f**. Human gut viromes, bovine viromes, porcine viromes, env viromes—unassembled viral metagenomes derived, respectively, from the human, bovine and porcine gut, or of non-host-associated environmental origin; human gut viromes (assem)—assemblies of human gut viral data sets; human gut whole, NH gut—whole community data sets derived from human or non-human gut, respectively; body, oral—whole community metagenomes from various human body sites or the oral cavity, respectively. env whole—whole community metagenomes non-host-associated environmental origin. Details of data sets in each group are provided in Supplementary Table S1

Ordination of all available data sets based on the ɸB124-14 relative abundance profile, generated a clear overall separation between viral metagenomes and those derived from whole communities (Fig. 3a, c, Supplementary Fig. S1 and Supplementary Table S2). Assembly of data sets was indicated to have only minimal impact on nMDS distributions based on ordination of assembled human gut viromes. These data sets displayed lower overall relative abundance values than unassembled counterparts, but collectively remained closely associated with unassembled data sets, and strongly separated from whole community metagenomes (Fig. 3a, c and Supplementary Fig. S1). When the relationship between viral data sets was examined in more detail, human gut viromes were observed to exhibit a clear and significant separation from other viral data sets (bovine, porcine and environmental) based on the ɸB124-14 relative abundance profile (Fig. 3b, c and Supplementary Fig. S1).

In contrast, ɸSYN5 ORF relative abundance profiles provided considerably poorer resolution of metagenome groups, and reduced the number of metagenome groups meeting minimum criteria for inclusion in this analysis (Fig. 3d, e, f and Supplementary Fig. S1). Use of the ɸSYN5 ecogenomic profile resulted in more highly dispersed groups, with less separation of viral data sets from each other, and from the whole community environmental metagenome group (Fig. 3d, e, f). A notable exception was an apparently enhanced ability to distinguish porcine and human gut-derived metagenomes with the ɸSYN5 profile (Fig. 3e, f). A comparable analysis using ɸKS10 was not possible due to the very low representation of sequences with homology to ɸKS10 ORFs in the majority of data sets.

### Use of the ɸB124-14 ecogenomic signature to identify human-associated pollution in environmental data sets

To evaluate the potential of the ɸB124-14 ecogenomic signature to identify the presence of human gut-associated pollution in environmental samples, we simulated the contamination of environmental viromes with human gut virome content. This was performed by adding the average human gut-derived relative abundance profile of ɸB124-14 to profiles obtained from environmental viral data sets. ɸB124-14 gut-associated profiles were added to environmental profiles at 'strengths' ranging from 100 to 0.01%, to explore the range over which the ɸB124-14 gut-associated ecogenomic signal may be detectable when combined with background environmental signals.

**Fig. 4:**
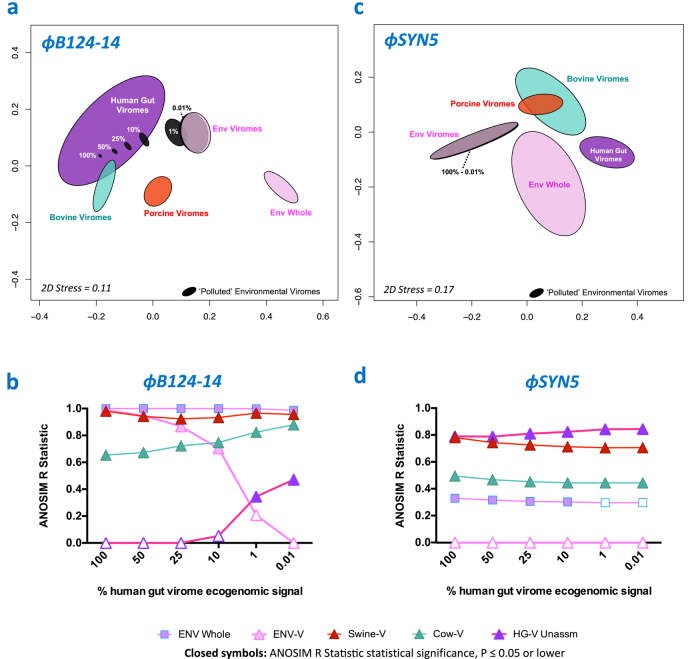
Detection of human gut-associated ecogenomic signals in simulated 'polluted' environmental data sets. The potential for the ɸB124-14 ecogenomic signal to identify human faecal pollution in environmental data sets was explored by simulating pollution of selected environmental viromes. This was achieved by combining average human gut virome ɸB124-14, or ɸSYN5 relative abundance profiles, with those of selected environmental viromes. Human gut-associated profiles were combined at 'strengths' ranging from 100 to 0.01% of human gut virome average, with profiles of viromes from the Bay of British Columbia, Sargasso Sea, Gulf of Mexico, Tampa Bay and Reclaimed Water. Relationships between groups of 'uncontaminated' and 'polluted' metagenomes were explored using nMDS and ANOSIM as for Fig. 3. **a**, **c** nMDS ordination of uncontaminated metagenomes and those modified to include either ɸB124-14 or ɸSYN5 human gut virome profiles. Filled ellipses show standard deviation of dispersion of each group relative to the group centroid. Black ellipse denotes groups of 'polluted' environmental data sets, with 'strength' (100–0.01%) of human gut signal added. **b**, **d** ANOSIM analysis of the differences between groups of metagenomes used in nMDS ordination. Charts show the ANOSIM R statistic for each uncontaminated group of metagenomes compared with data sets modified to simulate different levels of human faecal pollution. An increasing strength of separation between groups is indicated as the R statistic approaches 1 (total separation). Open symbols indicate no significant separation from the polluted data set compared, while closed symbols indicate significant separation (*P* ≤ 0.05). Human gut viromes, bovine viromes, porcine viromes, env viromes—unassembled viral metagenomes derived, respectively, from the human, bovine and porcine gut, or of non-host-associated environmental origin; env whole—whole community metagenomes non-host-associated environmental origin. Details of data sets in each group are provided in Supplementary Table S1

This showed a correlation between dilution of the ɸB124-14 human gut-associated ecogenomic signal, and separation of 'contaminated' data sets from human gut or 'uncontaminated' environmental viromes (Fig. 4a, b). As the ɸB124-14 ecogenomic signal strength decreased, contaminated data sets exhibited correspondingly increased separation from human gut viromes by nMDS and ANOSIM, and a closer association with uncontaminated environmental metagenomes (Fig. 4a, b). In addition, it is notable that contamination of environmental data sets with the human gut-derived ɸB124-14 ecogenomic signature also provided a clear indication of human gut-associated pollution specifically, and these data sets remained distinct and well separated from bovine and porcine viromes (Fig. 4a, b). In contrast, the same experiment using the ɸSYN5 human gut-derived relative abundance profile, provided no discernible separation of contaminated environmental data sets from uncontaminated viromes, in keeping with the alternative environmental ecogenomic signature exhibited by this phage, and reinforcing the gut-specific nature of the ɸB124-14 relative abundance profile across these data sets (Fig. 4c, d).

### Identification of human gut-associated genes in the ɸB124-14 genome

**Fig. 5:**
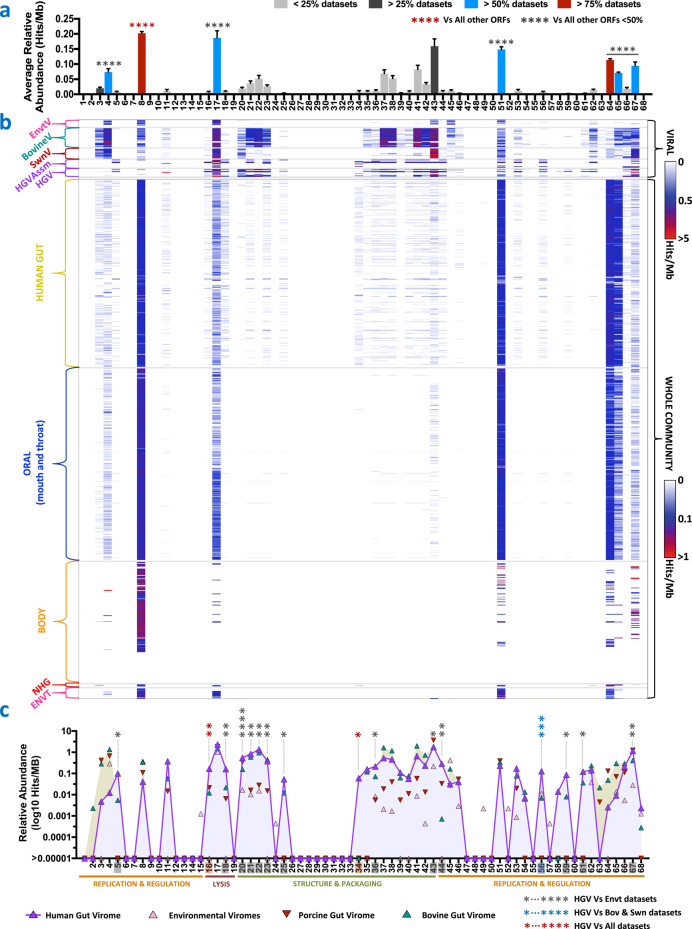
Identification of human gut-associated genes in the ɸB124-14 genome. The representation of each ɸB124-14 ORF in all data sets was used to assess the consistency of the human gut-associated ecogenomic signal across the phage genome, and identify ORFs with human gut affiliations. **a** Average relative abundance (hits/Mb), and representation of ɸB124-14 ORFS across all 840 data sets examined. Colours of bars indicate the % of data sets with at least one valid hit to each ORF as described in the associated legend. Significant differences in average relative abundance for ORFs represented in 50% of more of the data sets examined are shown by symbols above bars and colours indicate significance vs. all other ɸB124-14 ORFs, or significance vs. all other ɸB124-14 ORFs with less than 50% representation in data sets examined. Bars show SEM. **b** Heatmap showing relative abundance of individual ɸB124-14 ORFs in each metagenomic data set examined. Columns represent ORFs as indicated on **a** *x*-axis, and rows represent metagenomic data sets. The intensity of shading of each cell represented the relative abundance (hits/Mb) of each ORF in each particular metagenome, corresponding to the scale provided. **c** Relative representation of ɸB124-14 ORFs in human gut-derived viral data sets compared to other viromes. Points show the average relative abundance of each ORF in viral metagenomes from each category, expressed as Log_10_ hits/Mb. Membership of each ORF with previously described functional gene clusters in the ɸB124-14 genome [18] is indicated below the *x*-axis. Symbols above points indicate significantly greater relative abundance in human gut viromes compared with either all other viromes, or compared with those of environmental origin. *P < 0.05, **P < 0.01, ***P < 0.001, ****P < 0.0001. Details of data sets in each group are provided in Supplementary Table S1

To further delineate the human gut-associated ecogenomic signal inherent in ɸB124-14, and to identify genome regions with the strongest gut affiliation, we next explored the representation of individual ɸB124-14 ORFs in all metagenomes in more detail. This revealed that a subset of ɸB124-14 ORFs appear to exhibit a highly cosmopolitan distribution across ecosystems, with similar sequences in >50% of all data sets examined and representation in almost every habitat examined (Fig. 5a, b and Supplementary Table S3). These cosmopolitan ORFs are distributed throughout the ɸB124-14 genome and encode diverse functions including DNA recombination and repair, thymidylate synthase activity, peptidase activity and a phage anti-repressor, as well as ORFs of unknown function (Fig. 5a, b and Supplementary Table S3). The other phage genomes examined also contained examples of cosmopolitan ORFs, which were predicted to encode functions similar to counterparts in ɸB124-14 (Supplementary Figs. S2 and S3 and Supplementary Table S3).

This analysis also revealed a range of ORFs in the ɸB124-14 genome with a seemingly clear-cut human gut affiliation (Fig. 5b, c and Supplementary Table S4). These ORFs were relatively well represented in human gut viromes and human gut whole community data sets, as well as other mammalian gut viromes, but overall poorly represented in data sets from other habitats (Fig. 5b). These gut-associated ORFs were distributed throughout the ɸB124-14 genome, with a notable concentration in regions of the genome predicted to be involved in synthesis of the viral capsid and genome packaging (Fig. 5c [18]). When the representation of these gut-affiliated ɸB124-14 genomic regions was considered in viral data sets specifically, many were found to exhibit a significant enrichment in human gut viromes compared to environmental viromes, or in some cases all other viral data sets (Fig. 5c). In accordance with the other analyses conducted, no comparable human gut-associated pattern was observed for ɸSYN5 and ɸKS10 genomes, but ɸSYN5 ORFs were observed to be well represented in environmental data sets relative to other metagenomes examined (Supplementary Figs. S2 and S3).

### Simulation and modelling of virome-based source tracking using ɸB124-14 ecogenomic signatures

**Fig. 6:**
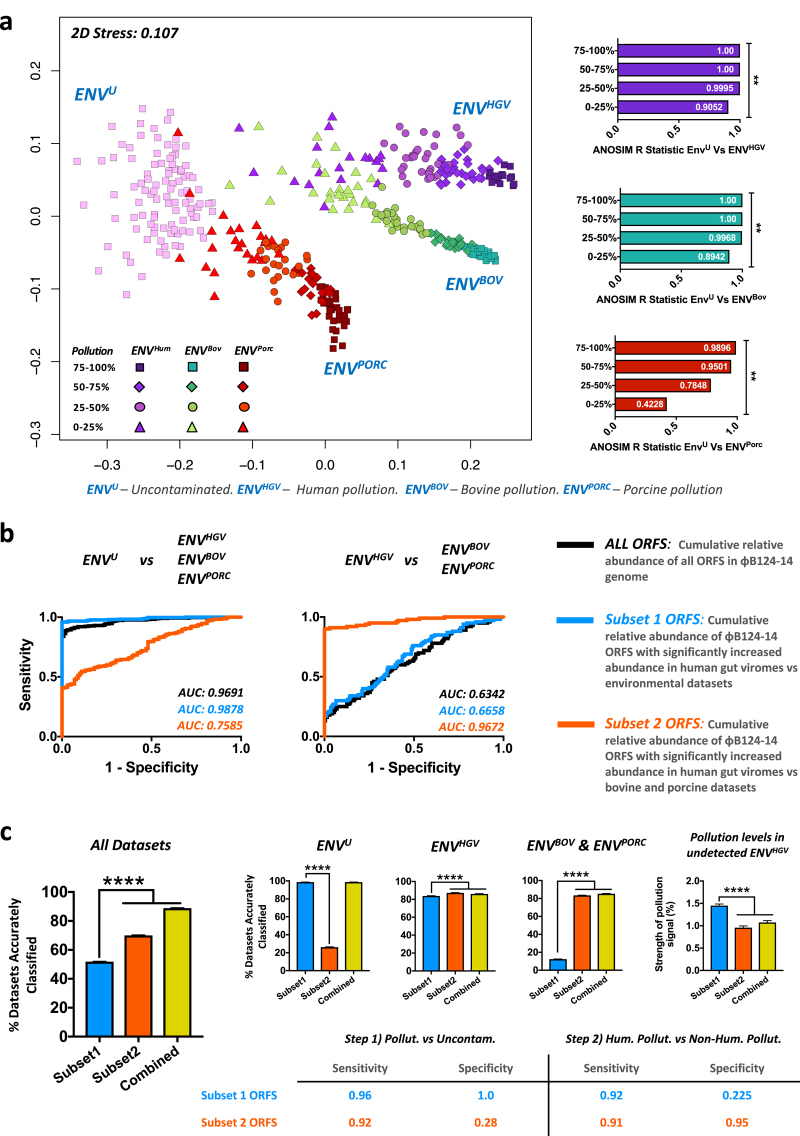
Simulation and modelling of virome-based source tracking using ɸB124-14 ecogenomic signatures. To evaluate the potential for the ɸB124-14 ecogenomic signature to be used in MST, we undertook more extensive Monte Carlo-based simulations of pollution using randomly permuted and polluted environmental viromes, and specific detection of human pollution using ɸB124-14 ORF relative abundance profiles. **a** nMDS and ANOSIM analysis of uncontaminated and 'polluted' permutations of environmental viral metagenomes. Symbol shape for polluted data sets (human, bovine or porcine) represents the strength of contamination as indicated by the associated key. ANOSIM shows the separation of groups of data sets with varying ranges of human or animal contamination, from uncontaminated environmental viromes (**P = 0.001). *ENV*^*U*^—uncontaminated environmental virome permutations; *ENV*^*HGV*^—environmental virome permutations contaminated by human gut ecogenomic signature; *ENV*^*BOV*^—environmental virome permutations contaminated by bovine gut ecogenomic signature; *ENV*^*PORC*^—environmental virome permutations contaminated by porcine gut ecogenomic signature; **b** ROC curves were constructed from randomly permuted and polluted data sets displayed in **a**, based on relative abundance profiles from all ɸB124-14 ORFS, or a subset of ORFS exhibiting significantly different  mean relative abundance in human gut viromes than other data sets (see Fig. 5c). Subset 1 ORFS = 5, 16, 18, 20, 21, 22, 23, 25, 34, 36, 43, 44, 59, 61 and 67; subset 2 ORFS = 16, 34 and 56. The area under curve (AUC) for each ROC curve indicate the diagnostic potential for cumulative relative abundance of each ORF combination to distinguish different groups of data sets, with values approaching 0.5 indicating little or no diagnostic power. All AUC were statistically significant at *P* ≤ 0.002. **c** Histograms show the proportion of data sets of each type (*ENV*^*U*^; *ENV*^*HGV*^; *ENV*^*BOV*^; *ENV*^*PORC*^) accurately identified by a two-step classification approach using threshold values indicative of either pollution in general (step 1) or human pollution more specifically (Step 2), selected based on sensitivity and specificity values generated by ROC analyses (a minimum sensitivity of 0.91). This pipeline was evaluated using threshold values for binning derived from either subset 1 ORFS, subset 2 ORFS or a combination in which subset 1 values were applied to step 1, and subset 2 values were applied to step 2. ****P < 0.0001. Error bars show standard error of the mean from 100 iterations with 100 new randomly permuted and polluted data sets of each type per iteration

To further probe the robustness of this habitat-related signal, and begin to provide insight into the potential sensitivity, specificity and accuracy of virome-based MST tools, we next simulated a more expansive and varied set of environmental viromes. This was achieved through random permutation of ecogenomic profiles derived from environmental data sets, followed by introduction of random levels of human, bovine or porcine pollution (based on addition of respective ɸB124-14 ecogenomic profiles). Ordination of these permuted and polluted data sets by nMDS indicated that the ɸB124-14 ecogenomic signal was still able to clearly segregate all groups of data, and in proportion to the strength of human, bovine or porcine signal applied (Fig. 6a). Data sets with lower levels of human and bovine pollution were also observed to converge, in keeping with previous analyses, but still remained clearly segregated from uncontaminated environmental data sets (Fig. 6a). Overall, this analysis suggested that the potential discriminatory power of the ɸB124-14 ecogenomic signal was preserved despite the additional wide variation in the innate background environmental signal, and that it could also distinguish different sources of pollution.

To evaluate the possible discriminatory power of ɸB124-14 relative abundance profiles and specific human gut-affiliated ORF subsets in more detail, ROC curves were constructed based on relative abundance profiles from all ɸB124-14 ORFs, as well as subsets exhibiting significantly higher representation in human gut viromes compared to other viral data sets (Fig. 6b). This revealed that the cumulative relative abundance profile derived from all ɸB124-14 ORFs had potentially high diagnostic potential in terms of distinguishing uncontaminated data sets from polluted environmental viromes, but held no real diagnostic potential for the distinction of human-polluted data sets from those subject to simulated bovine or porcine contamination (Fig. 6b). A comparable performance was also predicted when ROC analysis was based on ORFs with significantly increased mean relative abundance in human gut viromes compared to environmental viromes (designated subset 1; Fig. 6b). In contrast, ROC analysis based only on those ORFs exhibiting significantly higher average representation in human gut viromes compared to all other viromes analysed (designated subset 2; Fig. 6b), showed considerably greater potential for distinguishing data sets subject to human-derived pollution from non-human sources, but a reduced capacity for distinguishing polluted from unpolluted data sets in general (Fig. 6b). Collectively, these analyses indicated a two-step process utilising different ɸB124-14 ORF subsets should provide the best performance in terms of sensitivity, specificity and overall accuracy.

To test these predictions, threshold cumulative relative abundance values (minimum sensitivity of 0.91 and the highest available specificity) were selected from ROC analyses and applied to the two-step categorisation of randomly permuted and polluted data sets (Fig. 6c). In this process, data sets were first categorised as polluted or non-polluted (Step 1), and polluted data sets subsequently scrutinised further to identify those contaminated specifically with human-derived signals (Step 2). This experiment confirmed that relative abundance profiles from Subset 1 ORFs were able to distinguish polluted from unpolluted data sets with high accuracy (high sensitivity, high specificity), but performed poorly in subsequent specific identification of human-polluted data sets (high sensitivity, low specificity) (Fig. 6c). In contrast, the converse was observed for categorisation based solely on Subset 2 ORFs (Fig. 6c). However, a good overall performance was obtained when Subset 1 and Subset 2 relative abundance profiles were used in combination. The application of Subset 1 ORF profiles in Step 1, and Subset 2 ORF profiles in Step 2, resulted in a highly accurate distinction of polluted from unpolluted data sets, as well as specific identification of those contaminated by human-derived signatures (Fig. 6c).

## Discussion

Here we provide evidence that a distinctive, human gut-associated ecogenomic signature can extend to specific phage from the human gut virome and distinct ecogenomic signatures can be found in phage from other habitats. Our analysis, encompassing both viral and whole community metagenomic data sets covering a wide range of environments, reveals the existence of a clear human gut-associated ecogenomic signature within the *Bacteroides* ɸB124-14 genome [18]. Analysis of the representation of sequences with similarity to this phage genome clearly groups metagenomic data sets based on their environmental origin, and identified regions of the ɸB124-14 genome with the strongest human gut affiliation. Furthermore, through an in silico modelling approach, we provide preliminary proof-of-concept, and show these gut-associated genome regions likely hold sufficient discriminatory power for the development of phage-based metagenomic MST tools.

These findings are congruent with previous smaller-scale evaluations of the ɸB124-14 ecological profile using both sequence alignments [18], the tetranucleotide usage profile of the ɸB124-14 genome [25] and evaluation of phage replication in gut-specific host bacteria [19]. However, a notable difference in the present analysis was not only the increased scale, encompassing a considerably greater number and diversity of metagenomes than previous studies, but also the premise from which the ɸB124-14 genome was analysed.

We hypothesised that any gut-associated ecogenomic signature encoded by ɸB124-14 would be derived from the co-evolution of this phage and its bacterial host within the human gut, and should manifest as an increased relative abundance of sequences with similarity to ɸB124-14-encoded genes in viromes from this habitat. However, by default this gene-centric hypothesis also allows that not all ɸB124-14 genes would be subject to the same selective forces, or be expected to display the same levels of ecological success in a given viral community or host microbiome. Therefore, rather than a single unified and fixed genetic unit, we instead viewed ɸB124-14 as an assemblage of independent but associated genes, each with its own evolutionary trajectory within a given microbial community, and calculated representation in metagenomic data sets on an individual gene-by-gene basis. Exploration of the ɸB124-14 genome in this way is also more compatible with the mosaic nature and inherent plasticity of phage genomes [27–29], and stands to provide more flexibility in the use of phage sequences in the development of MST tools.

Overall, this approach allowed us to identify genes or genome regions with the strongest affiliation to the human gut microbiome in ɸB124-14, and therefore the most suitable potential targets for development of molecular or metagenome-based MST assays. Although only a general association with the mammalian gut virome (human, porcine and bovine) was initially noted in surveys of cumulative relative abundance, likely reflecting common features of these mammalian gut microbiomes (such as an abundance of *Bacteroides* sp. [30, 31]), discrete regions with more specific human gut affiliation were resolved through more detailed analysis of the ɸB124-14 genome. Importantly, our results also show this approach is equally capable of distinguishing alternative ecogenomic signatures in other phage, or indicating the absence of any habitat affiliation should clear ecogenomic signals not be readily identifiable in a phage genome.

This was clearly demonstrated by conducting identical analyses of phage from other environments (ɸSYN5 and ɸKS10), which are considered to have no notable association with the human gut microbiome, and displayed no human gut-related ecogenomic signature. A distinct environmental ecogenomic signature was detected in ɸSYN5 using this approach, while no discernible ecogenomic signal was apparent in ɸKS10. While ɸSYN5 observations are in keeping with the habitat of its bacterial host, the lack of any detectable ecological affiliation in ɸKS10 likely reflects the paucity of available data sets covering terrestrial habitats relevant to this bacteriophage, and the overall 'healthy' status of volunteers from which human metagenomes were derived. It is also possible that the temperate nature of ɸKS10 may contribute to the lack of a detectable ecogenomic profile, but the use of whole community metagenomes should compensate for this aspect of the ɸKS10 lifestyle. Collectively, analysis of both ɸSYN5 and ɸKS10 provide further support for the hypothesis that relative abundance profiles of genes similar to ɸB124-14 ORFs in metagenomic data sets are indeed reflective of a gut-related ecogenomic signal.

Congruent with the concept of ɸB124-14 as a collective of genes with independent evolutionary trajectories was the clear variability in gut affiliation of individual ORFs evident across the ɸB124-14 genome. Notably, no strong representation in any habitat was observed for some genes, while some aspects of the ɸB124-14 functional repertoire (the majority related to DNA regulation and replication) were indicated to be conserved across multiple disparate environments. Examples of similar highly cosmopolitan genes were also identified in ɸSYN5 and ɸKS10, and phage-encoded genes with broad environmental distribution have been reported in other studies [32–35], suggesting these may be relatively common within phage genomes. These cosmopolitan genes were counterbalanced by genes that showed a seemingly more provincial, gut-specific representation. Taken together, these observations are compatible with the notion that the abundance of genes similar to particular ɸB124-14 ORFs in human gut data sets reflects environmental selection on a gene-by-gene basis [36], the extant features of the human gut virome in terms of dominance of temperate phage and an intimate role for phage in community function and stability (reviewed in ref. [37]).

Using the ɸB124-14 relative abundance profile to 'contaminate' viral data sets of environmental origin, also permitted crude in silico simulations of human faecal pollution, and modelling of how MST tools based on bacteriophage ecogenomic profiles and gut-affiliated phage gene subsets may conceivably operate. In these experiments, we focused on viral metagenomes specifically due to the clear segregation of viromes in nMDS ordinations, and the proposed advantages of phage in MST applications [12, 13]. For initial evaluations (Fig. 4), the choice of environmental viral data sets 'polluted' was focused on those most likely to be already impacted by human activity and/or with a strong innate background environmental signal (e.g., temperate marine environments, coastal waters near major population centres and reclaimed water). The data sets selected therefore encompassed environmental viromes exhibiting the highest background ɸB124-14 cumulative relative abundance profiles, to provide a conservative and stringent evaluation of the potential for the ɸB124-14 gut-associated ecogenomic signal to distinguish polluted from uncontaminated environmental data sets. In addition, the degree to which the applied human-derived signal was diluted in these experiments was congruent with that observed for other indicators of pollution during events such as Combined Sewer Overflows [38, 39].

This evaluation demonstrated that the separation of polluted environmental data sets towards human gut viromes was in proportion to the strength of the introduced human gut-related ɸB124-14 signal. Expansion of this in silico modelling approach using a wider range of randomly permuted and polluted environmental profiles, and more focused ɸB124-14 ORF subsets indicated to have the greatest diagnostic power in ROC analyses, further demonstrated that the relative abundance of ɸB124-14 ORFs within different viromes can potentially distinguish those specifically contaminated with human-derived ecogenomic profiles with high accuracy. The levels of sensitivity and specificity achieved during these simulations were comparable to those reported for a wide range of qPCR-based methods using multiple or combined bacterial or viral gene targets (reviewed in ref. [3]).

Although the in silico modelling undertaken here affords only a very basic and simplistic simulation of pollution and the use of phage ecogenomic signatures for MST, these experiments nonetheless provide an initial proof-of-concept that viral metagenomic data sets can be distinguished in this way, and supports the possibility for development of new MST methods based on these concepts. Moreover, it should be noted that modelling undertaken here was based on only a single phage ecogenomic profile, and using only basic abundance thresholds to discriminate data sets. The metagenomic approach opens the potential to simultaneously utilise a large number of indicators derived from many phage, and move beyond simple abundance-based thresholds. The inclusion of further phage ecogenomic signatures, coupled with the development of more powerful diagnostic algorithms should further enhance performance of these approaches. Our use of different subsets of ɸB124-14 ORFs in distinct stages of data set categorisation during simulations, also serves to highlight some of the advantages of metagenomic approaches to MST.

Furthermore, unlike qPCR and other direct molecular biology assays, metagenomics can capture information on an almost unlimited array of genes present in a sample, as emphasis is placed on the analysis of sequence data to provide the actual diagnostic test. Because of this, once an initial metagenomic strategy for sampling and generation of sequence data has been developed, the cost, time and labour involved in continual adaptation and improvement of assays is considerably reduced. Modelling of new strategies is also readily implemented, and performance of multiple distinct algorithms or new 'tests' may be compared directly in parallel on the same samples and data sets, without compromising results of ongoing source tracking activities. This should provide considerable flexibility in the design, implementation and continued improvement of metagenome-based MST tools, and as new information and targets are identified these may be easily evaluated on historical data with established provenance, and incorporated into the MST pipeline without altering the basic sampling and sequencing protocols. It should also be noted that the generation of sequence data from samples is also no longer a major barrier to implementing such approaches. Fully portable and affordable sequencing platforms, such as the MinION from Oxford Nanopore Technologies, are commercially available, and have been used in the field for metagenomics analysis in habitats ranging from the Arctic Tundra to the International Space Station.

Nevertheless, care must be taken not to over interpret the results presented here, which should be considered in the context of the limitations and potential biases within existing metagenomic data sets, the relatively simplistic and crude modelling undertaken, as well as the relatively poor representation of most habitats afforded by the metagenomic data sets available. Metagenomes analysed here were drawn from a variety of sources, and vary in terms of construction methods, community coverage, assembly status, sample sizes and sample numbers. Because of this, the simple relative abundance approach used here intentionally employs more permissive criteria for identifying sequences with similarity to target sequences, to reduce the impact of these methodological variations and provide a conservative and robust comparison between data sets. This strategy seeks to identify general patterns in relative representation of broad functions between data sets rather than identical genes or sequences, with normalisation for differing depths of sequencing between data sets and has previously been shown to enable useful comparison of metagenomes generated by different approaches [25, 40–42]. Furthermore, the use of more permissive criteria in the relative abundance analyses were also intended to provide a more robust and conservative test of the phage ecogenomic signature hypothesis. In essence, these criteria should maximise the detection of conflicting non-specific signals in non-target data sets, meaning that distinct phage ecogenomic profiles need to be discernible against a higher level of background 'noise' to be identified in this analysis.

The utility of this approach was also supported in the present study, in which available data sets were shown to form cohesive and well-defined groups based on habitat in nMDS ordinations. Notable examples include conventional human gut metagenomes produced using distinct metagenomic techniques and sequencing methods [24, 43–46], which were clearly localised to a cohesive group. Comparison of assembled and unassembled versions of the same human gut viral data sets in these experiments also confirmed that assembly should have only minimal impact on the overall results obtained, and did not obscure the habitat-derived ecological signatures present in these metagenomes, or the distinction between viral and whole community data sets. Overall, available evidence suggests that the approaches we have used to compare data sets permit identification of genuine differences based on relative gene abundance and provide meaningful insight into habitat-associated features of these metagenomes.

Of more concern are the relatively small numbers of samples and data sets available for all habitats, most notably viromes and non-human gut whole community data sets. This is exacerbated by the high inter-individual variability noted in human viral metagenomes used here and in other studies [23, 25], but in practice for human gut viromes, this variation is likely to be offset to some degree by the fact that MST will be based on aggregate gut microbiome outputs from human populations as a whole, rather than individual microbiomes. However, a distinct geographic variation is also believed to exist in the human gut microbiome [18, 47, 48], and culture-based approaches utilising gut-associated phage infecting *Bacteroides* species have already highlighted the possible need to develop region-specific MST tools [3, 17]. Although here and in other studies, whole community human gut data sets derived from individuals from disparate geographic locations [43, 45, 46] were found to still group clearly based on habitat in higher-level analyses, the human gut viromes we analysed are derived exclusively from individuals residing in the United States, and so provide little insight into possible geographical effects. Moreover, the geographic variation in gut virome composition has yet to be subject to the same level of scrutiny directed towards the bacterial component of this ecosystem. In addition, the number of viral particles, derived levels of nucleic acids and details of sampling and processing methods that may provide a useful lower limit from which diagnostic relative abundance profiles can be calculated, remain to be determined. Further large-scale studies will be required to address these questions, fully test the hypotheses presented here and fully examine the potential for phage-based metagenomic MST tools derived from these ecogenomic concepts. This will not only entail the generation and use of a more extensive collection of viral metagenomes from relevant sources, but also the isolation and characterisation of further phage genomes from these habitats, including identification of those with ecogenomic signatures that may be utilised and incorporated into phage-based MST approaches.

In essence, the gene pool of a given microbial community adapts over time reflecting the challenges of life in a given habitat, as well as the ancestry of community members [49]. Here we provide evidence that this may also manifest as a bias within the viral gene pool of particular microbiomes, forming the basis for a habitat-related ecogenomic signature, which can also be detected in individual member phage. Overall, the work presented here provides new fundamental insights into phage ecology that could support the development of a novel range of highly specific, sensitive, rapid and portable phage-based metagenomic MST tools.

## Methods

### Cumulative relative abundance of genes with similarity to phage-encoded ORFs

The representation of sequences with similarity to phage-encoded functions and calculation of cumulative relative gene representation between data sets was performed as previously described [18, 40, 50], but with the following modifications: unassembled viral data sets were surveyed by mapping raw sequencing reads to translated ɸB124-14, ɸSYN5 or ɸKS10 ORFs using BlastX. Assembled whole community metagenomes and assembled viral data sets were searched using tBlastn with amino acid sequences from each predicted phage ORF. For both data set types, valid hits were considered to be those generating ≥35% identity over ≥50% of the query sequence and an *e*-value of ≤1e^−5^. Valid hits were used to calculate the relative abundance of each phage-encoded ORF in each data set (expressed as Hits/Mb of sequence data). The cumulative relative abundance of ORFs encoded by each phage was taken as the sum of all individual ORF relative abundances. Blast searches and calculation of relative abundance were automated using a custom PERL script (access and support is freely available on request to authors), which implemented BLAST v2.2.29 with default settings, searched custom Blast databases generated from each metagenomic data set, processed BLAST outputs to identify valid hits based on criteria above and calculated relative abundance for each phage ORF in each metagenomic data set. Data were saved as *.csv format files and imported into Microsoft XL for further analysis. Significant differences in cumulative relative abundances between metagenomes were assessed using the Kruskall–Wallis test with Dunn’s correction for multiple comparisons. Statistical analyses and generation of scatterplots were performed in GraphPad Prism 6.0 for Mac OS X.

### Unsupervised ordination of metagenomic data sets based on phage-related ecogenomic profiles

Ordination of metagenomes was performed using the Vegan package (v2.4) [51] in R to conduct nMDS [26] and ANOSIM [26], using the *metaMDS* and *anosim* functions, respectively. For nMDS and ANOSIM, individual gene relative abundance profiles for each phage in each metagenomic data set (calculated as described above) were used and only data sets exhibiting sequences with similarity to at least two distinct ORFs per phage (i.e., a minimum of two valid hits to distinct ORFs in BLAST searches) were included. Relative abundance data were square root transformed, before being used to construct Bray–Curtis distance matrices (Vegan package in R), and then for nMDS (with a minimum of 1000 random starts). Square root transformed data were used directly without further processing for ANOSIM analyses, which calculated the level and significance of separation between defined groups of metagenomes based on habitat of origin. The ANOSIM R statistic indicates increasing separation of groups as values approach 1, while statistical significance is provided by an associated *P* value. Graphical representations of nMDS ordinations were produced using Vegan ordiplot functions in R. ANOSIM data were visualised using GraphPad Prism 6.0 for OS X.

### In silico simulation of human faecal pollution in environmental data sets

Contamination of environmental data sets with human pollution was simulated by addition of the ɸB124-14 human gut virome ecogenomic signature to selected environmental viromes. The average relative abundance of each ɸB124-14 ORF within human gut viromes [24] (*n* =12) was added to the corresponding ɸB124-14 ORF relative abundance in selected environmental viromes on a gene-by-gene basis, at 'strengths' ranging from 100 to 0.01%. The viromes subjected to this simulated human faecal pollution were selected based on those most likely to be already impacted by human activity, and/or contain a strong innate background environmental signal distinct from that of the gut microbiome (Bay of British Columbia, Sargasso Sea, Gulf of Mexico, Tampa Bay and Reclaimed Water). The ability of ɸB124-14 human gut ecogenomic signals to discriminate polluted environmental data sets from original uncontaminated data sets was evaluated using nMDS ordination and ANOSIM, as described above.

### Identification of regions of the ɸB124-14 with the strongest ecogenomic signal

The variation in the 'strength' of the human gut-associated ɸB124-14 ecogenomic signal across the phage genome and representation in data sets from distinct environmental groups was assessed by transforming all relative abundance values by addition of a small positive value (y + 0.00001), before conversion to Log_10_ hits/MB DNA. Differences in relative abundance within human gut viromes or ɸB124-14 ORFs was compared to profiles observed in bovine and porcine gut viromes, environmental viromes, as well as whole community human gut and environmental metagenomes. Significant differences between the relative representation of ɸB124-14 ORFs in human gut viromes compared to other data sets was determined using the Kruskall–Wallis test with Dunn’s correction for multiple comparisons, in GraphPad Prism 6 for OS X.

### Simulation and modelling of virome-based source tracking using ɸB124-14 ecogenomic signatures

The use of ɸB124-14 relative abundance profiles for microbial source tracking was evaluated using a Monte Carlo-based simulation with uniform probability distribution input, derived from the maximum baseline relative abundance values for each ɸB124-14 ORF across all environmental viral metagenomes. In these simulations, permutations of environmental ɸB124-14 relative abundance profiles were generated through random variation of each ORF relative abundance value, ranging from 0 to the maximum value observed for a given ORF across all environmental viromes. Copies of randomly permuted environmental viromes were subsequently subjected to simulated in silico pollution through addition of average human, bovine or porcine ɸB124-14 relative abundance profiles, at randomly selected signal strengths ranging from 0 to 100%. In each iteration, 100 randomly permuted environmental viromes were created and used to generate 100 randomly polluted data sets of each type (human, bovine and porcine). Data from a single iteration was used to visualise relationships between data sets using nMDS and ANOSIM as described for unsupervised ordination of metagenomic data sets above, and also to construct ROC curves based on cumulative relative abundance profiles for either all ORFs, or subsets found to be significantly increased in relative abundance compared to other data sets (see Fig. 5c). Data from all iterations were used to evaluate the performance of cumulative relative abundance thresholds in accurately identifying human-polluted data sets in a two-step binning process, based on threshold values derived from ROC analyses. Step 1 was used to categorise data sets as either polluted or non-polluted. In Step 2, data sets categorised as polluted in Step 1 were sorted further into ‘human-polluted’ and ‘non-human polluted’ categories, using a second threshold value from ROC analyses. Threshold values were selected to achieve the best possible sensitivity and specificity, but with a minimum sensitivity of 0.91. ROC analysis and statistical comparisons of performance of ORF combinations in categorising data sets (ANOVA with Bonferroni correction) were conducted using GraphPad Prism for OS X.

## Electronic supplementary material

Supplementary Information

## References

[1] Ahmed W, Hughes B, Harwood V (2016). Current status of marker genes of *Bacteroides* and related taxa for identifying sewage pollution in environmental waters. Water.

[2] Griffith JF, Cao Y, McGee CD, Weisberg SB (2009). Evaluation of rapid methods and novel indicators for assessing microbiological beach water quality. Water Res.

[3] Harwood VJ, Staley C, Badgley BD, Borges K, Korajkic A (2014). Microbial source tracking markers for detection of fecal contamination in environmental waters: relationships between pathogens and human health outcomes. FEMS Microbiol Rev.

[4] Leclerc H, Mossel DA, Edberg SC, Struijk CB (2001). Advances in the bacteriology of the coliform group: their suitability as markers of microbial water safety. Annu Rev Microbiol.

[5] Haack SK, Fogarty LR, Wright C (2003). *Escherichia coli* and enterococci at beaches in the Grand Traverse Bay, Lake Michigan: sources, characteristics, and environmental pathways. Environ Sci Technol.

[6] Ishii S, Yan T, Shively DA, Byappanahalli MN, Whitman RL, Sadowsky MJ (2006). *Cladophora* (*Chlorophyta*) spp. harbor human bacterial pathogens in nearshore water of Lake Michigan. Appl Environ Microbiol.

[7] McLellan SL, Salmore AK (2003). Evidence for localized bacterial loading as the cause of chronic beach closings in a freshwater marina. Water Res.

[8] Whitman RL, Shively DA, Pawlik H, Nevers MB, Byappanahalli MN (2003). Occurrence of *Escherichia coli* and enterococci in *Cladophora* (*Chlorophyta*) in nearshore water and beach sand of Lake Michigan. Appl Environ Microbiol.

[9] Tan B, Ng C, Nshimyimana JP, Loh LL, Gin KYH, Thompson JR (2015). Next-generation sequencing (NGS) for assessment of microbial water quality: current progress, challenges, and future opportunities. Front Microbiol.

[10] Gómez-Doñate M, Casanovas-Massana A, Muniesa M, Blanch AR (2016). Development of new host-specific *Bacteroides* qPCRs for the identification of fecal contamination sources in water. Microbiologyopen.

[11] Knights D, Kuczynski J, Charlson ES, Zaneveld J, Mozer MC, Collman RG (2011). Bayesian community-wide culture-independent microbial source tracking. Nat Methods.

[12] Gómez-Doñate M, Payán A, Cortés I, Blanch AR, Lucena F, Jofre J (2011). Isolation of bacteriophage host strains of *Bacteroides* species suitable for tracking sources of animal faecal pollution in water. Environ Microbiol.

[13] Lee JE, Lim MY, Kim SY, Lee S, Lee H, Oh HM (2009). Molecular characterization of bacteriophages for microbial source tracking in Korea. Appl Environ Microbiol.

[14] Harwood VJ, Boehm AB, Sassoubre LM, Vijayavel K, Stewart JR, Fong TT (2013). Performance of viruses and bacteriophages for fecal source determination in a multi-laboratory, comparative study. Water Res.

[15] Jofre J, Blanch AR, Lucena F, Muniesa M (2014). Bacteriophages infecting *Bacteroides* as a marker for microbial source tracking. Water Res.

[16] McMinn BR, Korajkic A, Ashbolt NJ (2014). Evaluation of *Bacteroides fragilis* GB-124 bacteriophages as novel human-associated faecal indicators in the United States. Lett Appl Microbiol.

[17] Payan A, Ebdon J, Taylor H, Gantzer C, Ottoson J, Papageorgiou GT (2005). Method for isolation of *Bacteroides* bacteriophage host strains suitable for tracking sources of fecal pollution in water. Appl Environ Microbiol.

[18] Ogilvie LA, Caplin J, Dedi C, Diston D, Cheek E, Bowler L (2012). Comparative (meta)genomic analysis and ecological profiling of human gut-specific bacteriophage φB124-14. PLoS ONE.

[19] Ebdon J, Muniesa M, Taylor H (2007). The application of a recently isolated strain of Bacteroides (GB-124) to identify human sources of faecal pollution in a temperate river catchment. Water Res.

[20] Pope WH, Weigele PR, Chang J, Pedulla ML, Ford ME, Houtz JM (2007). Genome sequence, structural proteins, and capsid organization of the cyanophage Syn5: a ‘horned’ bacteriophage of marine Synechococcus. J Mol Biol.

[21] Goudie AD, Lynch KH, Seed KD, Stothard P, Shrivastava S, Wishart DS (2008). Genomic sequence and activity of KS10, a transposable phage of the *Burkholderia cepacia* complex. BMC Genome.

[22] Seed KD, Dennis JJ (2005). Isolation and characterization of bacteriophages of the *Burkholderia cepacia* complex. FEMS Microbiol Lett.

[23] Minot S, Sinha R, Chen J, Li H, Keilbaugh SA, Wu GD (2011). The human gut virome: Inter-individual variation and dynamic response to diet. Genome Res.

[24] Reyes A, Haynes M, Hanson N, Angly FE, Heath AC, Rohwer F (2010). Viruses in the faecal microbiota of monozygotic twins and their mothers. Nature.

[25] Ogilvie LA, Bowler LD, Caplin J, Dedi C, Diston D, Cheek E (2013). Genome signature-based dissection of human gut metagenomes to extract subliminal viral sequences. Nat Commun.

[26] Clarke K (1993). Non-parametric multivariate analyses of changes in community structure. Aust J Ecol.

[27] Hatfull GF, Hendrix RW (2011). Bacteriophages and their genomes. Curr Opin Virol.

[28] Hendrix RW, Smith MC, Burns RN, Ford ME, Hatfull GF (1999). Evolutionary relationships among diverse bacteriophages and prophages: all the world’s a phage. Proc Natl Acad Sci USA.

[29] Brüssow H, Canchaya C, Hardt WD (2004). Phages and the evolution of bacterial pathogens: from genomic rearrangements to lysogenic conversion. Microbiol Mol Biol Rev.

[30] Looft T, Allen HK, Cantarel BL, Levine UY, Bayles DO, Alt DP (2014). Bacteria, phages and pigs: the effects of in-feed antibiotics on the microbiome at different gut locations. ISME J.

[31] Jami E, Israel A, Kotser A, Mizrahi I (2013). Exploring the bovine rumen bacterial community from birth to adulthood. ISME J.

[32] Breitbart M, Miyake JH, Rohwer F (2004). Global distribution of nearly identical phage-encoded DNA sequences. FEMS Microbiol Lett.

[33] Manrique P, Bolduc B, Walk ST, van der Oost J, de Vos WM, Young MJ (2016). Healthy human gut phageome. Proc Natl Acad Sci USA.

[34] Breitbart M, Rohwer F (2005). Here a virus, there a virus, everywhere the same virus?. Trends Microbiol.

[35] Roux S, Brum JR, Dutilh BE, Sunagawa S, Duhaime MB, Loy A (2016). Ecogenomics and potential biogeochemical impacts of globally abundant ocean viruses. Nature.

[36] Brito IL, Yilmaz S, Huang K, Xu L, Jupiter SD, Jenkins AP (2016). Mobile genes in the human microbiome are structured from global to individual scales. Nature.

[37] Ogilvie LA, Jones BV (2015). The human gut virome: a multifaceted majority. Front Microbiol.

[38] Madoux-Humery AS, Dorner S, Sauvé S, Aboulfadl K, Galarneau M, Servais P (2013). Temporal variability of combined sewer overflow contaminants: evaluation of wastewater micropollutants as tracers of fecal contamination. Water Res.

[39] Passerat J, Ouattara NK, Mouchel JM, Rocher V, Servais P (2011). Impact of an intense combined sewer overflow event on the microbiological water quality of the Seine River. Water Res.

[40] Jones BV, Begley M, Hill C, Gahan CGM, Marchesi JR (2008). Functional and comparative metagenomic analysis of bile salt hydrolase activity in the human gut microbiome. Proc Natl Acad Sci USA.

[41] Jones BV, Sun F, Marchesi JR (2010). Comparative metagenomic analysis of plasmid encoded functions in the human gut microbiome. BMC Genome.

[42] Ogilvie LA, Jones BV (2012). Dysbiosis modulates capacity for bile acid modification in the gut microbiomes of patients with inflammatory bowel disease: a mechanism and marker of disease?. Gut.

[43] Kurokawa K, Itoh T, Kuwahara T, Oshima K, Toh H, Toyoda A (2007). Comparative metagenomics revealed commonly enriched gene sets in human gut microbiomes. DNA Res.

[44] Nelson KE, Weinstock GM, Highlander SK, Worley KC, Creasy HH, Wortman JR (2010). A catalog of reference genomes from the human microbiome. Science.

[45] Qin J, Li R, Raes J, Arumugam M, Burgdorf KS, Manichanh C (2010). A human gut microbial gene catalogue established by metagenomic sequencing: commentary. Nature.

[46] Gill SR, Pop M, Deboy RT, Eckburg PB, Turnbaugh PJ, Samuel BS (2006). Metagenomic analysis of the human distal gut microbiome. Science.

[47] Suzuki TA, Worobey M. Geographical variation of human gut microbial composition. Biol Lett. 2014;10:20131037. 10.1098/rsbl.2013.1037 PMC3949373 24522631

[48] Yadav D, Ghosh TS, Mande SS (2016). Global investigation of composition and interaction networks in gut microbiomes of individuals belonging to diverse geographies and age-groups. Gut Pathog.

[49] Ley RE, Peterson Da, Gordon JI (2006). Ecological and evolutionary forces shaping microbial diversity in the human intestine. Cell.

[50] Jones BV (2010). The human gut mobile metagenome: a metazoan perspective. Gut Microbes.

[51] Oksanen J, Guillaume Blanchet F, Friendly M, Kindt R, Legendre P, McGlinn D, Minchin PR, O’Hara RB, Simpson GL, Solymos P, Stevens MH, Szoecs EWH. Vegan: community ecology package. R package version. 2016;2:4–0. https://cran.r-project.org/package=vegan.

