# Supplementary material for: Resolution of habitat-associated ecogenomic signatures in bacteriophage genomes and application to microbial source tracking
Source: ISME J. 2017 Dec 19;12(4):942–58. doi: 10.1038/s41396-017-0015-7 (PMC5864186; doi:10.1038/s41396-017-0015-7)
Supplement: Supplementary file 1 — Supplementary Information [file 41396_2017_15_MOESM1_ESM.pdf]

Supplementary Information for Ogilvie *et al.*,

"Resolution of habitat-associated ecogenomic signatures in bacteriophage genomes and application to microbial source tracking"

Supplementary Table S1: Overview of datasets and sequences utilised.

Dataset type	Habitat/Sequence type	Source[1]	Reference/comment
**Viral Metagenomes**	Human Gut **	NCBI SRA	(Reyes *et al.*, 2010)
	Swine gut	CAMERA	(Allen *et al.*, 2011)
	Reclaimed Water	CAMERA	(Rosario *et al.*, 2009)
	Tampa Bay	CAMERA	(McDaniel *et al.*, 2008)
	Sargasso Sea, Bay of British Columbia, Gulf of Mexico, Arctic Ocean	CAMERA	(Angly *et al.*, 2006)
	Marine Virus metagenome	CAMERA	Gordon and Betty Moore Foundation Marine Microbiology Initiative. Sequenced at the Broad Institute: http://www.broadinstitute.org/annotation/viral/Phage/Home.html
	Limpolar Lake	CAMERA	(López-Bueno *et al.*, 2009)
	Rice Paddy Soil Viruses **	NCBI	(Kim *et al.*, 2008)
	Marine Saltern	CAMERA	(Dinsdale *et al.*, 2008)
	Stromolite	CAMERA	(Desnues *et al.*, 2008)
	Viral Spring	CAMERA	(Schoenfeld *et al.*, 2008)
	Bovine	Cyverse iMicrobe	http://imicrobe.us/
	Cow	Cyverse iMicrobe	http://imicrobe.us/
**Whole Community Metagenomes**	Human Gut (MetaHit) – Danish, Spanish (n=124)	EMBL	(Qin *et al.*, 2010) http://www.bork.embl.de/~arumugam/Qin_et_al_2010/
	Human Gut – Japanese (n=13)	CAMERA	(Kurokawa *et al.*, 2007)
	Human Gut – American (n=2)	CAMERA	(Gill *et al.*, 2006)

	NIH Human Microbiome Project (All body sites)	NIH	(Nelson *et al.*, 2010) http://hmpdacc.org/. Accessed Feb 2014
	Canine Gut	CAMERA	(Swanson *et al.*, 2011)
	Global Ocean Sampling Expedition	CAMERA	(Yooseph *et al.*, 2007; Rusch *et al.*, 2007)
	Waseca County Farm Soil	CAMERA	(Tringe *et al.*, 2005)
	Termite Gut	CAMERA	(Warnecke *et al.*, 2007)
	Acid Mine Drainage	CAMERA	(Tyson *et al.*, 2004)
	Washington Lake	CAMERA	(Kalyuzhnaya *et al.*, 2008)
	Marine Metagenome	CAMERA	Gordon and Betty Moore Foundation Marine Microbiology Initiative. Sequenced at the Broad Institute: http://www.broadinstitute.org/annotation/viral/Phage/Home.html
	Mouse Gut	CAMERA	(Turnbaugh *et al.*, 2006)
	Whale Fall	CAMERA	(Tringe *et al.*, 2005)
	Chicken Caecum	CAMERA	(Qu *et al.*, 2008)
**Phage Genomes**	B124-14	EMBL	(Ogilvie *et al.*, 2012)
	KS10	NCBI	(Goudie *et al.*, 2008)
	SYN5	NCBI	(Pope *et al.*, 2007)

[1] Datasets and genome sequences utilised in this project were obtained from a range of publically accessible repositories:

CAMERA (Sun *et al.*, 2011): Community Cyberinfrastructure for Advanced Microbial Ecology Research and Analysis.

CAMERA Homepage: https://portal.camera.calit2.net/gridsphere/gridsphere**.** Datasets now available from Cyverse iMicrobe: http://imicrobe.us/

NCBI: National Centre for Biotechnology Information (http://www.ncbi.nlm.nih.gov).

NCBI SRA: Pyrosequencing reads generated from virus-like particles by (Reyes *et al.*, 2010) were obtained from the NCBI short read archive, project SRA012183 (http://www.ncbi.nlm.nih.gov/sra).

EMBL: Metagenomes comprising the MetaHIT dataset (Qin *et al.*, 2010) were obtained from the European Molecular Biology Laboratory database *via* the link provided in the table.

HMP: NIH Human Microbiome Project **(**http://hmpdacc.org/)

Supplementary Table S2: ANOSIM analyses for non-human viromes vs whole community datasets in nMDS analysis (Figure 3a, Supplementary Figure 1a).

	**Environmental viromes***	**Bovine viromes***	**Porcine viromes***
**Human Gut Whole**	0.9666 (0.001)	0.9966 (0.001)	0.9865 (0.001)
**Human Oral**	0.9703 (0.001)	0.9996 (0.001)	0.9914 (0.001)
**Human Body**	0.7377 (0.001)	0.9655 (0.001)	0.9318 (0.001)
**Non-human gut**	0.8784 (0.004)	0.9998 (0.001)	1.0 (0.001)
**Environmental Whole**	0.8654 (0.001)	0.9999 (0.001)	1.0 (0.001)

* Values provide ANOSIM R scores for non-human viral metagenomes (columns) compared with whole community datasets (rows). Figures in parentheses indicate the P value and statistical significance of each R value.

Supplementary Table S3: Functional assignment of cosmopolitan ϕB124-14 ORFs

Distribution	ORF[1]	Predicted Function	Putative product
**Cosmopolitan ORFs** homologues in >50% of datasets	**ϕB124-14**		
	**4**	DNA replication and regulation	**Putative essential recombination protein** (*Bacteroides* phage B40-8018; YP_002221556.1)
	**8**	DNA replication and regulation	**ThyA, Thymidylate synthase** (*Bacteroides* phage B40-8, B40-8016; YP_002221554.1). Complete ThyA (pfam00303; TIGR3284) and pyrimidine synthase/hydroxymethylase conserved domains detected (cd00351)
	**17**	Lysis	**Putative peptidase** (*Bacteroides* phage B40-8, B40-8010) YP_002221548.1). Complete conserved domains from M15_3 type peptidase detected (pfam08291) and partial domains from M15_2 and uncharacterised bacterial proteins detected (pfam05951 and COG3108 respectively)
	**51**	DNA replication and regulation	**Putative single-strand DNA binding protein** (*Bacteroides* phage B40-8, B40-8032; YP_002221570.1). Complete SSB_OBF conserved domains detected (cd04496) and partial single stranded binding protein domains detected (pfam00436;TIGR00621;COG0629)
	**64**	DNA replication and regulation	**Putative mismatch repair protein**. Similar to hypothetical protein PARMER_02659 from *Parabacteroides merdae* ATCC 43184 (ZP_02032642.1). Partial MutS mismatch repair conserved domains detected (pfam01624, PRK05399, TIGR01070,COG0249)
	**65**	DNA replication and regulation	**Putative resolvase/recombinase**. Similar to multiple promoter invertase from *Bacteroides* sp. 3_2_5 (ZP_04840901.1), and resolvase from *Bacteroides ovatus* SD CC 2a (ZP_06722395.1). Complete domains from serine recombinase type proteins (PRK13413, pfam00239,cd03768,smart00857), and Helix_turn_helix Hin_like domains detected (cd00569)
	**67**	DNA replication and regulation	**Putative phage anitrepressor**. Similar to antirepressor from *Lactobacillus* phage Lrm1 (YP_002117696.1), *L. rhamnosus* Lc 705m (YP_003173532.1) and Bacteriodes phage B40-8 (B40-8022, YP_002221560.1). Complete phage_pRha regulatory domain (pfam09669) and ANT KilAC domain detected (pfam03374)
	**ϕSYN5**		
	**30**	DNA replication and regulation	**gp30. Protein coding gene of unknown function**. Within the DNA replication and regulation functional module of the phage genome
	**ϕKS10**		
	**23**	DNA replication and regulation	**DNA binding protein HU-beta**. Conserved domains detected: DNA binding (dc13831, pfm00216, COG0776); histone like domain (smart00411), provisional transcriptional regulator HU sub-unit beta, integration host factor (TIGR00987) and the DNA sequence specific (IHF) and non-specific (HU) domains (cl00257)

[1]ORF numbers and functional assignments correspond to those represent on genetic maps of the ϕB124-14 genome shown in (Ogilvie *et al.*, 2012).

ORF – Open Reading Frame

Supplementary Table S4: Functional assignment of human gut-specific ϕB124-14 ORFs

Distribution		ORF[1]	Predicted Function	Putative product
**Human gut-specific ORFs (ϕB124-14)** (significantly ***increased*** relative abundance	HG viromes vs all datasets	**16**	Lysis	Hypothetical protein (*Bacteroides* phage B40-8, B40-8011; YP_002221549.1)
	HG viromes vs all viromes	**34**	Structure & Packaging	No significant hits
	HG viromes vs bovine & swine datasets	**56**	DNA replication and regulation	Hypothetical protein (*Bacteroides* phage B40-8, B40-8028; YP_002221543.1)

[1]ORF numbers and functional assignments correspond to those represent on genetic maps of the ΦB124-14 genome shown in (Ogilvie *et al.*, 2012).

ORF – Open Reading Frame

Supplementary Figure 1

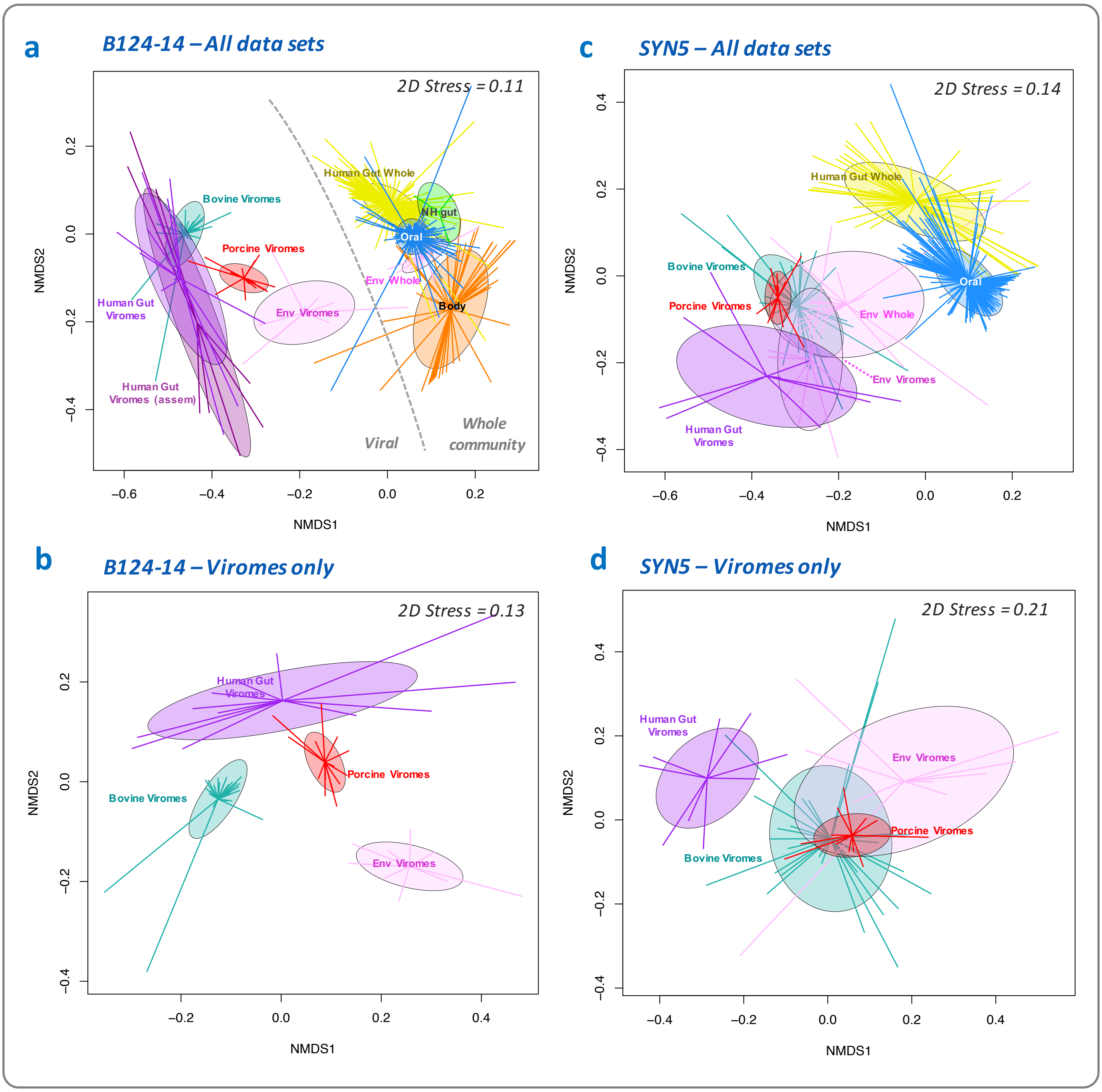

Supplementary Figure S1: Segregation of metagenomic datasets based on the ϕB124-14 ecogenomic signature. The ability to differentiate metagenomes from distinct habitats based on bacteriophage ecogenomic signals was explored using non-metric multidimensional scaling (nMDS) and Analysis of Similarities (ANOSIM) between groups. Ordination of datasets by nMDS was performed based on relative abundance profiles of all individual ORFs in phage genomes. Metagenomic datasets were classified by broad environmental origin, and individual datasets generating less than 2 valid hits to any phage ORFs were excluded from this analysis. **a-d)** nMDS ordination of metagenomes based on ϕB124-14 or ϕSYN5 ORF relative abundance profiles. Raw data was subject to square root transformation before being used to construct Bray-Curtis similarity matrices, and visualised using nMDS plots. Ellipses show standard deviation of dispersion of each group relative to the group centroid. Lines within groups/ellipses show distance of individual data points in each group relative to the group centroid. Group numbers relate to the associated legend which provides the broad environmental origin of datasets in each group. See also Figure 3.

Supplementary Figure 2

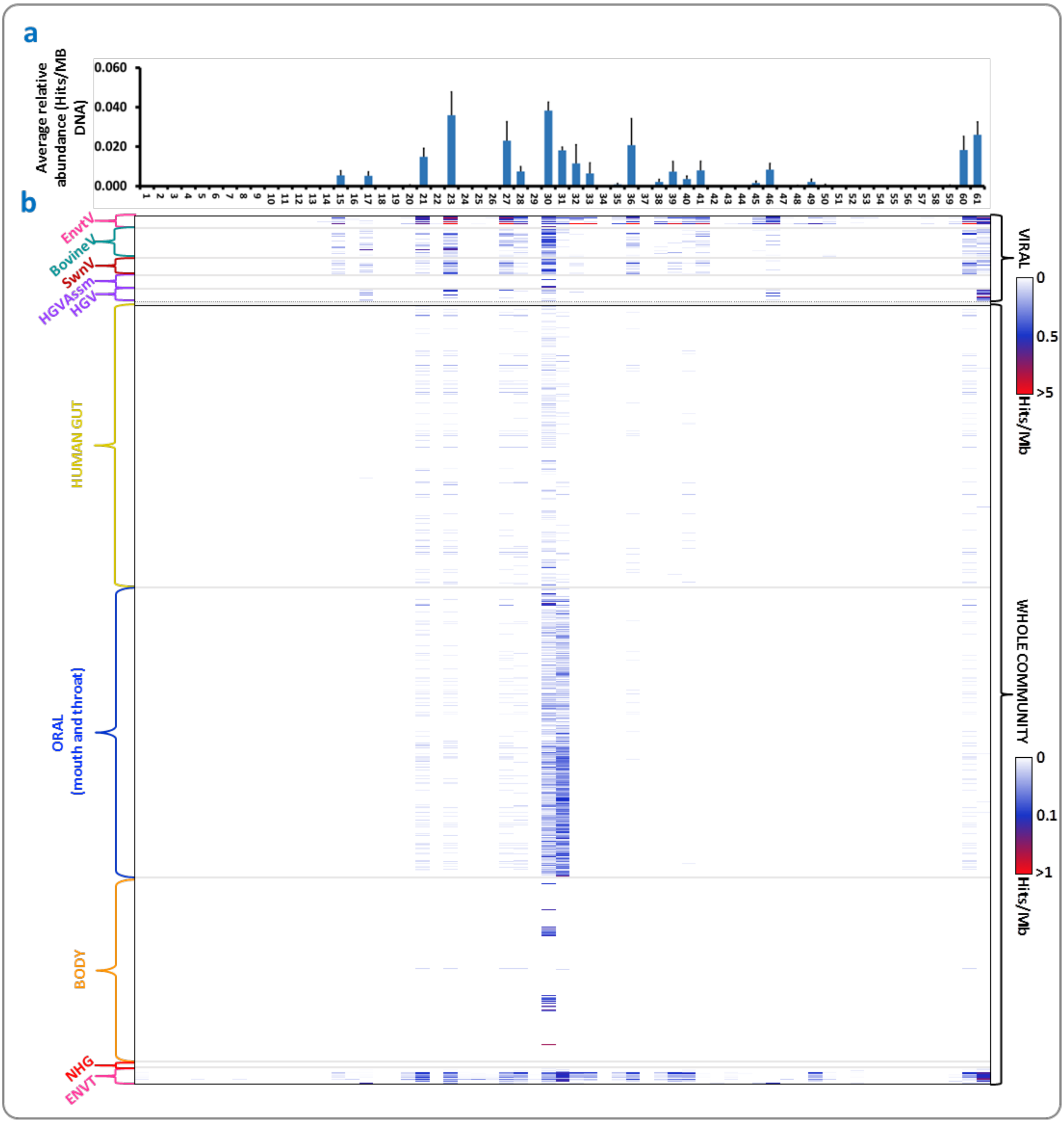

Supplementary Figure S2: Relative abundance of ΦSYN5 encoded functions within metagenomic datasets of diverse origin. Both assembled and unassembled datasets were used in this analysis (See Supplementary Table S1). The relative representation of ϕSYN5 ORFs calculated based on valid BlastX read mapping to translated ϕSYN5 ORFS for unassembled datasets (≥35% identity ≥50% query coverage, ≤ $1e^{-5}$), or valid tBlastn hits in assembled datasets with translated ϕB124-14 ORFs as query sequences (≥35% identity ≥50% query coverage, ≤ $1e^{-5}$). Relative representation of ϕSYN5 ORFs in all datasets was expressed in Hits/Mb a) Average relative abundance, and representation of ϕSYN5

ORFs across all 860 datasets examined. Bars show SEM. b) Heatmap showing relative abundance of individual ϕSYN5 ORFs in each individual metagenomic dataset examined. Columns represent ORFs as indicated on Part (a) X-axis, and rows represent metagenomic datasets. The broad category into which each dataset has been grouped is also provided. The Intensity of shading of each cell represented the relative abundance of that ORF in a particular metagenome, corresponding to the scale provided for viral and whole community datasets. EnvtV – Viral metagenomes derived from, non-host associated (marine, freshwater, soil, wastewater); SwnV – Viral metagenomes derived from porcine gut; HGV – Unassembled viral metagenomes derived from the human gut; HGVAssm – Assemblies of human gut viromes; HUMAN GUT– Whole community metagenomes derived from human stool samples; ORAL– whole community metagenomes derived from the human mouth or throat; BODY– whole community metagenomes derived from a range of non-gut human body sited (nares, vagina, skin). NHG – Whole community metagenomes from non-human gut habitats (canine, insect, murine); ENVT – Whole community dataset derived from non-host associated environments (soil, marine, freshwater).

Supplementary Figure 3

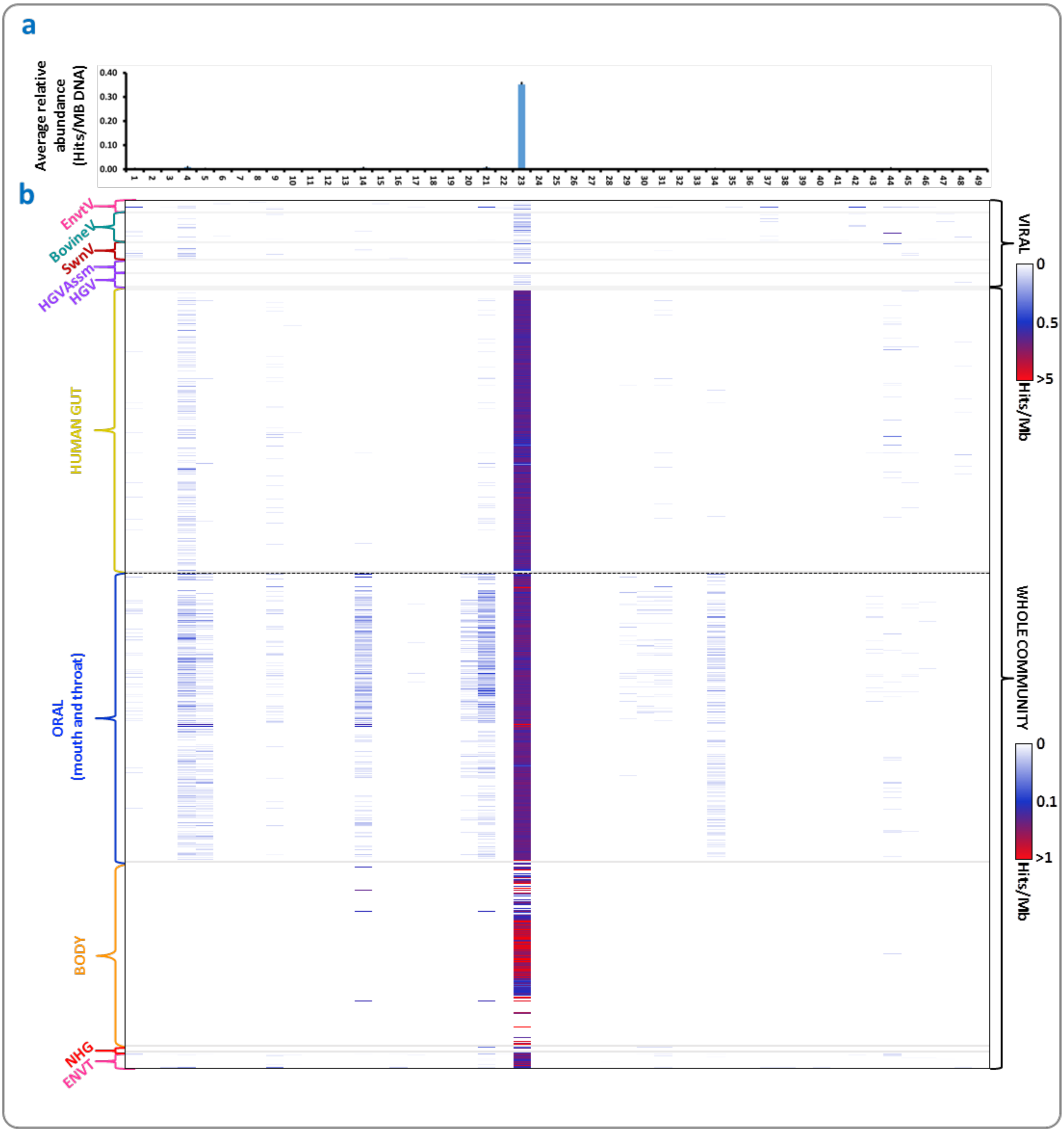

Supplementary Figure S3: Relative abundance of ΦKS10-encoded functions within metagenomic datasets of diverse origin. Both assembled and unassembled datasets were used in this analysis (See Supplementary Table S1). The relative representation of ϕKS10 ORFs calculated based on valid BlastX read mapping to translated ϕKS10 ORFs for unassembled datasets (≥35% identity ≥50% query coverage, ≤ $1e^{-5}$), or valid tBlastn hits in assembled datasets with translated ϕKS10 ORFs as query sequences (≥35% identity ≥50% query coverage, ≤ $1e^{-5}$). Relative representation of ϕKS10 ORFs in all datasets was expressed in Hits/Mb **a)** Average relative abundance, and representation of ϕKS10

ORFs across all 860 datasets examined. Bars show SEM. **b)** Heatmap showing relative abundance of individual ϕKS10 ORFs in each individual metagenomic dataset examined. Columns represent ORFs as indicated on Part **(a)** X-axis, and rows represent metagenomic datasets. The broad category into which each dataset has been grouped is also provided. The Intensity of shading of each cell represented the relative abundance of that ORF in a particular metagenome, corresponding to the scale provided for viral and whole community datasets. EnvtV – Viral metagenomes derived from, non-host associated (marine, freshwater, soil, wastewater); SwnV – Viral metagenomes derived from porcine gut; HGV – Unassembled viral metagenomes derived from the human gut; HGVAssm – Assemblies of human gut viromes; HUMAN GUT– Whole community metagenomes derived from human stool samples; ORAL– whole community metagenomes derived from the human mouth or throat; BODY– whole community metagenomes derived from a range of non-gut human body sited (nares, vagina, skin). NHG – Whole community metagenomes from non-human gut habitats (canine, insect, murine); ENVT – Whole community dataset derived from non-host associated environments (soil, marine, freshwater).

Supplementary References

Allen HK, Looft T, Bayles DO, Humphrey S, Levine UY, Alt D, *et al.* (2011). Antibiotics in feed induce prophages in swine fecal microbiomes. *MBio* **2**. e-pub ahead of print, doi: 10.1128/mBio.00260-11.

Angly FE, Felts B, Breitbart M, Salamon P, Edwards RA, Carlson C, *et al.* (2006). The marine viromes of four oceanic regions. *PLoS Biol* **4**: e368.

Desnues C, Rodriguez-Brito B, Rayhawk S, Kelley S, Tran T, Haynes M, *et al.* (2008). Biodiversity and biogeography of phages in modern stromatolites and thrombolites. *Nature* **452**: 340–343.

Dinsdale E a, Edwards R a, Hall D, Angly F, Breitbart M, Brulc JM, *et al.* (2008). Functional metagenomic profiling of nine biomes. *Nature* **452**: 629–32.

Gill SR, Pop M, Deboy RT, Eckburg PB, Turnbaugh PJ, Samuel BS, *et al.* (2006). Metagenomic analysis of the human distal gut microbiome. *Science* **312**: 1355–9.

Goudie AD, Lynch KH, Seed KD, Stothard P, Shrivastava S, Wishart DS, *et al.* (2008). Genomic sequence and activity of KS10, a transposable phage of the Burkholderia cepacia complex. *BMC Genomics* **9**: 615.

Kalyuzhnaya MG, Lapidus A, Ivanova N, Copeland AC, McHardy AC, Szeto E, *et al.* (2008). High-resolution metagenomics targets specific functional types in complex microbial communities. *Nat Biotechnol* **26**: 1029–34.

Kim K-H, Chang H-W, Nam Y-D, Roh SW, Kim M-S, Sung Y, *et al.* (2008). Amplification of uncultured single-stranded DNA viruses from rice paddy soil. *Appl Environ Microbiol* **74**: 5975–85.

Kurokawa K, Itoh T, Kuwahara T, Oshima K, Toh H, Toyoda A, *et al.* (2007). Comparative metagenomics revealed commonly enriched gene sets in human gut microbiomes. *DNA Res* **14**: 169–81.

López-Bueno A, Tamames J, Velázquez D, Moya A, Quesada A, Alcamí A. (2009). High diversity of the viral community from an Antarctic lake. *Science* **326**: 858–61.

McDaniel L, Breitbart M, Mobberley J, Long A, Haynes M, Rohwer F, *et al.* (2008). Metagenomic analysis of lysogeny in Tampa Bay: implications for prophage gene expression. Butler G (ed). *PLoS One* **3**: e3263.

Nelson KE, Weinstock GM, Highlander SK, Worley KC, Creasy HH, Wortman JR, *et al.* (2010). A Catalog of Reference Genomes from the Human Microbiome. *Science (80-)* **328**: 994–999.

Ogilvie LA, Caplin J, Dedi C, Diston D, Cheek E, Bowler L, *et al.* (2012). Comparative (meta)genomic analysis and ecological profiling of human gut-specific bacteriophage φB124-14. *PLoS One* **7**: e35053.

Pope WH, Weigele PR, Chang J, Pedulla ML, Ford ME, Houtz JM, *et al.* (2007). Genome Sequence, Structural Proteins, and Capsid Organization of the Cyanophage Syn5: A 'Horned' Bacteriophage of Marine Synechococcus. *J Mol Biol* **368**: 966–981.

Qin J, Li R, Raes J, Arumugam M, Burgdorf KS, Manichanh C, *et al.* (2010). A human gut microbial gene catalogue established by metagenomic sequencing: Commentary. *Nature* **464**: 28.

Qu A, Brulc JM, Wilson MK, Law BF, Theoret JR, Joens LA, *et al.* (2008). Comparative Metagenomics Reveals Host Specific Metavirulomes and Horizontal Gene Transfer Elements in the Chicken Cecum Microbiome Ahmed N (ed). *PLoS One* **3**: e2945.

Reyes A, Haynes M, Hanson N, Angly FE, Heath AC, Rohwer F, *et al.* (2010). Viruses in the faecal microbiota of monozygotic twins and their mothers. *Nature* **466**: 334–338.

Rosario K, Nilsson C, Lim YW, Ruan Y, Breitbart M. (2009). Metagenomic analysis of viruses in reclaimed water. *Environ Microbiol* **11**: 2806–2820.

Rusch DB, Halpern AL, Sutton G, Heidelberg KB, Williamson S, Yooseph S, *et al.* (2007). The Sorcerer II

Global Ocean Sampling Expedition: Northwest Atlantic through Eastern Tropical Pacific Moran NA (ed). *PLoS Biol* **5**: e77.

Schoenfeld T, Patterson M, Richardson PM, Wommack KE, Young M, Mead D. (2008). Assembly of viral metagenomes from yellowstone hot springs. *Appl Environ Microbiol* **74**: 4164–74.

Sun S, Chen J, Li W, Altintas I, Lin A, Peltier S, *et al.* (2011). Community cyberinfrastructure for Advanced Microbial Ecology Research and Analysis: the CAMERA resource. *Nucleic Acids Res* **39**: D546-51.

Swanson KS, Dowd SE, Suchodolski JS, Middelbos IS, Vester BM, Barry KA, *et al.* (2011). Phylogenetic and gene-centric metagenomics of the canine intestinal microbiome reveals similarities with humans and mice. *ISME J* **5**: 639–649.

Tringe SG, von Mering C, Kobayashi A, Salamov AA, Chen K, Chang HW, *et al.* (2005). Comparative metagenomics of microbial communities. *Science* **308**: 554–7.

Turnbaugh PJ, Ley RE, Mahowald MA, Magrini V, Mardis ER, Gordon JI. (2006). An obesity-associated gut microbiome with increased capacity for energy harvest. *Nature* **444**: 1027–131.

Tyson GW, Chapman J, Hugenholtz P, Allen EE, Ram RJ, Richardson PM, *et al.* (2004). Community structure and metabolism through reconstruction of microbial genomes from the environment. *Nature* **428**: 37–43.

Warnecke F, Luginbühl P, Ivanova N, Ghassemian M, Richardson TH, Stege JT, *et al.* (2007). Metagenomic and functional analysis of hindgut microbiota of a wood-feeding higher termite. *Nature* **450**: 560–5.

Yooseph S, Sutton G, Rusch DB, Halpern AL, Williamson SJ, Remington K, *et al.* (2007). The Sorcerer II Global Ocean Sampling Expedition: Expanding the Universe of Protein Families Eddy S (ed). *PLoS Biol* **5**: e16.
